# Flexible and Conductive Bioelectrodes Based on Chitosan-Carbon Black Membranes: Towards the Development of Wearable Bioelectrodes

**DOI:** 10.3390/nano11082052

**Published:** 2021-08-12

**Authors:** Mireia Buaki-Sogó, Laura García-Carmona, Mayte Gil-Agustí, Marta García-Pellicer, Alfredo Quijano-López

**Affiliations:** 1 Instituto Tecnológico de la Energía (ITE), Avenida Juan de la Cierva 24, 46980 Paterna, Spain; mayte.gil@ite.es (M.G.-A.); marta.garcia@ite.es (M.G.-P.); alfredo.quijano@ite.es (A.Q.-L.); 2 Instituto de Tecnología Eléctrica, Universitat Politècnica de València, Camino de Vera s/n Edificio 6C, 46022 Valencia, Spain

**Keywords:** carbon black, chitosan, conductive membrane, biocompatible electrodes, glucose sensor, glucose biofuel cell, wearable sensing

## Abstract

Wearable sensors for non-invasive monitoring constitute a growing technology in many industrial fields, such as clinical or sport monitoring. However, one of the main challenges in wearable sensing is the development of bioelectrodes via the use of flexible and stretchable materials capable of maintaining conductive and biocompatible properties simultaneously. In this study, chitosan-carbon black (CH-CB) membranes have been synthesized using a straightforward and versatile strategy and characterized in terms of their composition and their electrical and mechanical properties. In this sense, CH-CB membranes showed good conductivity and mechanical resistance thanks to the presence of carbon black, which decreases the insulating behavior of chitosan, while flexibility and biocompatibility are maintained due to the dual composition of the membrane. Thus, flexible and biocompatible conductive bioelectrodes have been developed by the combined use of CH and CB without the use of toxic reagents, extra energy input, or long reaction times. The membranes were modified using the enzymes Glucose Oxidase and Laccase in order to develop flexible and biocompatible bioelectrodes for enzymatic glucose biofuel cells (BFCs) and glucose detection. A BFC assembled using the flexible bioelectrodes developed was able to deliver 15 µW cm^−2^, using just 1 mM glucose as biofuel, and up to 21.3 µW·cm^−2^ with higher glucose concentration. Additionally, the suitability of the CH-CB membranes to be used as a glucose sensor in a linear range from 100 to 600 µM with a limit of detection (LOD) of 76 µM has been proven. Such demonstrations for energy harvesting and sensing capabilities of the developed membrane pave the way for their use in wearable sensing and energy harvesting technologies in the clinical field due to their good mechanical, electrical, and biocompatible properties.

## 1. Introduction

The use of wearable devices is currently receiving a great deal of attention from the scientific community, society, and industry [1,2]. Wearable devices are based on the use of wireless strategies for real time and non-invasive monitoring in many applications, with special relevance in health and fitness personalized monitoring. There are many types of wearable devices, including smartwatches, wristbands, hearing aids, electronic/optical tattoos, head-mounted displays, subcutaneous sensors, electronic footwear, and electronic textiles [3]. In this context, new trends in wearable devices lead to self-powered sensors that use energy harvesting approaches to obtain clean energy from biofluids without impacting the environment. Thus, approaches such as enzymatic biofuel cells (BFCs) are extremely suitable to power this technology because they are able to produce energy by using molecules such as the glucose or lactate present in sweat, blood, or interstitial fluid [4,5].

In this sense, either for sensors, BFC development, or wearable electronics, specific requirements regarding body adaptation and biocompatibility of the materials are greatly needed for the efficient applicability of this technology so that it can be used by humans in a continuous way without losing their conductive and catalytic properties. Traditionally, non-miniaturized electronics was the most common approach to develop bioelectrodes for biosensors and BFC. However, they present low resistance to deformation and, therefore, flexible materials come into play in the new trends for wearable development due to their superior mechanical properties. To achieve the targeted mechanical stability, flexible electrodes require the introduction of features such as stretchability in order to endure mechanical strain during regular use, or biocompatible features in order to enable their use in human tissues [6]. This fact is especially important in the case of wearable and implantable devices because they should allow for mechanical deformations and be in continuous contact with the user. Hence, it is essential to use non-toxic and biocompatible materials to minimize the risk of epithelization or allergic reactions. In most wearable and implantable devices, such as biosensors and BFCs, enzymes are included as an active redox catalyst; therefore, biocompatibility of the bioelectrode materials is again an essential requirement, not only for their human usage, but also to maintain enzyme stability.

Conductive polymers such as polyaniline (PANI), polypyrrole (PPy), polythiophene (PT), and polyethylenedioxide thiophene (PEDOT) have been widely used for bioelectrode development due to their biocompatibility and conductivity and the presence of tunable functionalities [7,8,9,10]. However, their application in biodevices is limited for different reasons. In the case of PANI, it is one of the most common conductive polymers used in biosensors. Conductivity can reach 100 S/cm when doped and presents good selectivity, sensitivity, and biocompatibility; however, the polymer is not active at neutral pH, which hinders its application for bioelectrodes development. PPY is another common conductive polymer used in the development of bioelectrodes. It presents a good ability for composite formation and it is biocompatible, but it is fragile, and the synthesis procedure often involves toxic reagents. PT has good optical properties, and conductivity can reach 10 S/cm if doped. PT is biocompatible if co-polymerized but presents a low mechanical integrity, which prevents its application in biosensing or for energy harvesting purposes. Finally, PEDOT is one of the most promising conductive polymers for bioelectrode development because it is biocompatible and presents good solubility properties if doped with PSS in order to avoid swelling and collapse in aqueous solutions. When doped with PSS, conductivity can reach up to 200 S/cm [7,8,9,10]. But PEDOT synthesis usually involves the use of toxic reagents and complex procedures that are difficult to implement out of the lab scale.

Due to the above-mentioned limitations of conductive polymers for bioelectrode development, polymers from biological origin appear to be a very interesting alternative to overcome solubility and biocompatibility issues. This is due to their similar film and membrane forming abilities and their ideal biocompatibility. Biopolymers are usually derived from cellulose, and they include chitosan (CH), alginate, or carrageenan, among others. CH, despite being a natural insulator, presents interesting properties such as flexible film forming ability, low toxicity, biocompatibility, biodegradability, antibacterial activity, and hydrophilicity [11]. However, its main drawback in being used for bioelectrode development is the fact that it does not present conductive properties. Nevertheless, insulating polymers with satisfactory mechanical properties has already been used to develop conductive substrates by the addition of materials such as graphite, carbon black (CB), carbon nanotubes (CNTs), or metallic particles such as colloidal silver or gold [12,13]. Thus, electrically conductive polymer composites, including biopolymers, have attracted a great deal of interest due to the combination of simultaneous flexibility and conductivity [8].

In this sense, carbon black (CB) has gained attention due to its suitable properties in terms of conductivity and biocompatibility for bioelectrode development in sensing applications [14,15] or reinforcing filler for biological matrices [16]. CB is a low-cost nanomaterial (c.a. 1 EUR/Kg), frequently used in the development of low-cost electrochemical devices. CB displays specific properties such as high surface area, sphere diameter of nanometrical size, and high I_D_/I_G_ ratio. It also exhibits excellent electrical conductivity, is dispersible in many solvents, and can be functionalized, and presents defect sites and fast electron transfer kinetics [17]. CB has also been used as an additive in many polymers to enhance physical properties including electrostatic discharge protection, electromagnetic shielding, or thermal and mechanical resistance [18]. Regarding CB suitability for on-body use, only a few studies regarding CB biocompatibility have been found [19,20,21,22,23,24]. In this situation, the only risk associated with CB is found when it comes from CB particle inhalation and indoor exposure as opposed to skin or tissue contact because particle exposure could lead to the appearance of toxicity effects in the lung; cardiovascular disease; or malfunction, neurotoxicity, or carcinogenity [20,21]. Although biocompatibility experiments have not been carried out in this work, all the individual components used for the synthesis and that are present in the final CH-CB membrane are well reported to be non-toxic and biocompatible. Specifically, incubation of HeLa cells with carbon nanomaterials and chitosan in a well stablished MTT cell viability assay where the role of chitosan to decrease the cytotoxicity of carbon nanomaterials has been proven. Furthermore, PC12 cell biocompatibility studies using chitin and carbon nanomaterials exhibited no cytotoxicity in the MTT assays [22,23]. The expected applicability of the developed membrane is for it to be used in contact with the skin. In this sense, it is worth mentioning that commercially available tattoo inks are currently composed of carbon black, and in several studies reported in the literature, CB is actually used as a control for biological safety evaluations of other carbon particles, which strongly support the assumption of non-toxicity of the two materials used for the development of the CH-CB membrane [19,24].

In order to explore the benefits provided by the use of a CH and CB combination for wearable sensing technology, different techniques have been reviewed to create biocompatible nanomaterial-based membranes. In this sense, several examples of CH doped with carbon-based materials have been proven to have suitable conductivity properties and the ability to be shaped in the form of films, electrospun membranes, and porous structured materials such as supports for the development of bioelectrodes, wound healing, and tissue repair [25,26,27,28,29]. In these approaches, the CH-carbon doped structured materials have presented conductivity values ranging from 0.0002 to 0.25 S∙cm^−1^, as is the case of a structured hybrid membrane composed of CH and graphene oxide, MWCNT, or CB [16,30,31,32,33]. Regarding CB-based conductive films, systems found in the literature include an electrospun CH-CB membrane with 62.5% *w/w* of CB with respect to CH with ideal mechanical properties, high conductivity, and chemical stability [34]; the use of CH together with polypirrole for tissue engineering applications [35]; or the use of CB to increase conductivity in a polyuretane film [18]. However, the former approaches involve the use of additional reagents and/or binders or solvents as is the case of PVDF or acetone and NMP for the development of the previous slurry for immunosensors [15] and techniques more complex than knife casting such as electrospinning for the preparation of self-standing chitosan-carbon black membranes [34].

In this study, flexible and biocompatible conductive bioelectrodes have been developed by the combined use of CH and CB to improve electron transfer ability and minimize insulating capacity. However, there are important differences between the membranes developed in this study and previously reported membranes regarding simplicity and sustainability of the synthesis method that will not need any toxic reagents and solvents, extra energy input, or long reaction times. In addition, the CH-CB membranes developed in this study could be easily sized and shaped to suit any application, which makes them highly suitable materials for bioelectrode development, along with their ability to host specific enzymes due to their chemical and biocompatible properties.

Additionally, it has been proven that the flexible and biocompatible CH-CB membranes synthesized in this study can be used as bioelectrodes for anode and working electrode development for BFCs and biosensors, respectively. Thus, energy harvesting and glucose detection have been tested in order to test their suitability for further wearable device development.

## 2. Materials and Methods

### 2.1. Reagents and Instrumentation

For the synthesis of the CH-CB membranes, CH of medium molecular weight with a deacetylation degree >85% (Sigma–Aldrich. St. Louis, MO, USA), glacial acetic acid reagent grade, NaOH (Scharlab, S.L., Barcelona, Spain), and C65 Carbon black powder (Nanographi, Ankara, Turkey) were used. Distilled water was used as a solvent.

For enzyme immobilization, Glucose oxidase (GOx) from Aspergillus niger Type VII and Laccase from Trametes versicolor were obtained from Sigma–Aldrich (St. Louis, MO, USA). N-(3-dimethylaminopropyl)-N-ethylcarbodiimide (EDC) was purchased from Iris Biotech Gmbh (Marktredwitz, Germany), and N-hydroxysulfosuccinimide (NHS) was provided by Sigma Aldrich (St. Louis, MO, USA). 2-(N-morpholino) ethanesulfonic acid for the MES buffer and D-Glucose biotechnology grade standard was provided by VWR LIFE SCIENCE (Radnor, PA, USA). For the preparation of Phosphate Buffer solutions, 0.1 M Na_2_HPO_4_ and NaH_2_PO_4_ from VWR LIFE SCIENCE (Radnor, PA, USA) were used. Planetary Ball Mill Retsch PM100 (Retsch GmbH, Dusseldorf, Germany) was used for the preparation of the slurry for the CH membranes. An orbital shaker JP Selecta Rotaterm (JP Selecta, Barcelona, Spain) was used for the washing stage in the membrane preparation. A Heidenhain-METRO MT 1281 length gauge (Heidenhain GmbH, Traunreut, Germany) was used to control membrane thickness.

### 2.2. CH-CB Membrane Fabrication and Enzyme Immobilization

CH (2% *w/w*) was stirred at room temperature in an aqueous solution of acetic acid 0.3 M overnight. It was then sonicated over 1 h in an ultrasound bath in order to remove air bubbles from the solution. The CH solution in acetic acid was placed in a ball milling jar, adding CB in order to achieve the desired weight percentage of 25%, 75%, 100% and 200%. CB was manipulated using a suitable protective mask to avoid particle inhalation. As an alternative, the slurry components could also be mixed and prepared inside a glove box. Ball milling was performed over 30 min, and the slurry obtained was recovered and cast onto a glass surface with the aid of a casting knife. The cast film was dried at room temperature overnight. Once the membrane was dry, it was neutralized by spraying a NaOH 1M solution on the surface. The membrane was removed from the glass surface and underwent a washing procedure using distilled water and was performed in an orbital shaker at 80 rpm over 10 min. The washing stage was repeated three times until a neutral pH was obtained in the washing medium. The wet membrane was dried at room temperature by fixing it onto a flat surface overnight. Five different membranes of each composition were developed in order to check the reproducibility of the process and the variability in the electrical properties of the developed membranes.

For bioelectrode development, the CH-CB membrane was cut to a geometric area of 0.6 cm^2^ and attached to a cupper film to create the electric contact (electroactive surface of 1.2 cm^2^). The cupper was then fully isolated to avoid interferences during measurement, leaving just the CH-CB membrane exposed.

GOx and Laccase were then immobilized in the membranes for bioelectrode development. In the case of the GOx-based anode and the working electrode, 10 mg/mL GOx was immobilized in the CH-CB membranes using a covalent approach based on the use of EDC/NHS. In due course, GOx, NaOH 0.1 M was used to oxidize the CH-CB membranes electrochemically by amperometry at 1.2 V for 300 s. EDC/NHS 36 mM and 17 mM, respectively, were then used in the MES buffer at pH 6 to react with the carboxylic-acid groups and form the amine-reactive intermediate before adding 10 mg/mL of GOx dissolved in the saline buffer (PBS) [36,37]. In the case of Laccase, it was adsorbed onto the CH-CB membrane by contacting a 10 mg/mL solution of Laccase in PB pH 6.5 for 30 min. 1 mL of EDC 36 mM and 1 mL of NHS 17 mM in PB pH 6.5 were added to this solution, and the reaction was allowed to proceed for 2 h at room temperature. The membrane bioelectrode was then washed using distilled water and kept at 4 °C until use [38,39,40].

### 2.3. Characterization Methods

The four-point probe method was used in order to determine the electrical features of the CH-CB membranes. A Signatone S302 stand equipped with a SP4 probe head of Tungsten Carbide tips, provided by Microworld (Microworld^®^, Grenoble, France), was used to measure the sheet resistance. The device was connected to a Keithley 2410 Source Measurement Unit (Keithley, Cleveland, OH, USA) to record the collected data of intensity and voltage. From this data, sheet resistance (ρ) in Ω sq^−1^ can be obtained by applying the formula ρ = 4.532 × V/I, where 4.532 is a correction factor for the equipment given by the manufacturer. From the value of the sheet resistance, the electrical conductivity (σ) can be calculated using the formula σ = 1/Rs, where Rs is the resistivity of the material in Ω cm. Rs is obtained from the product of sheet resistance (ρ) measured using the 4-point method (in Ω sq^−1^) by the measured sample thickness.

Focused Ion Beam–Field Emission Scanning Electron Microscopy (FIB–FESEM) was performed using Zeiss Auriga Compact equipment at a voltage of 1.5 kV.

Analysis of the mechanical properties of the membranes was carried out using a DEBEN microassay module to analyze micromechanical properties. The membranes to be tested were cut into 1.5 cm × 3 cm fragments. The module was configured with a force of 2000 N. The membrane was subjected to a determined tensile force, and elongation of the sample was recorded until breakage, along with the consequent drop in the applied force.

X-ray Photoelectron Spectroscopy (XPS) spectra were acquired on a Kratos AXIS Supra spectrometer (Kratos, Manchester, UK) with a monochromatic Al KR X-ray source, using pass energy of 20 eV for regions and 160 eV for wide spectra. To provide a precise energy calibration, the XPS binding energies were referenced to the C1s peak at 284.9 eV.

An Autolab PGSTAT 12 potentiostat (Eco Chemie, Utrecht, The Netherlands) and a Zhaner IM6 electrochemical workstation provided with a PP241 module for high currents was used for electrochemical impedance spectroscopy, cyclic voltammetry, chronoamperometry, and BFC polarization curve determination. Electrochemical characterization of the GOx bioanode, Laccase biocathode, and working electrode for the glucose sensor were performed in a three electrode configuration using the developed membrane-based bioelectrodes as the working electrode, a reference electrode of Ag/AgCl, and a platinum wire as the counter electrode. Electrochemical impedance spectroscopy was performed in a 5 mM K^3+/4+^[Fe (CN)_6_] in PB solution, pH 7.5 at a potential of 0.3 V, and an amplitude of 10 mV. Cyclic voltammetry was performed at a scan rate of 4 mV s^−1^ and 75 mV s^−1^ for bioanode evaluation and at 10 mV s^−1^ in the case of the Laccase biocathode. Chronoamperometry was performed at 0 and −0.45 V for biocathode and glucose biosensor evaluation, respectively. For the BFC polarization curve, the GOx bioanode and Laccase biocathode were then used together in a two-electrode configuration at 5 mV s^−1^. The I-V curve was recorded in the potential range of 0 to 0.4 V. The working electrolyte was PB pH 7 for CV and CA analysis and biofuel cell polarization curve determination.

## 3. Results

### 3.1. Fabrication and Characterization of Flexible Chitosan-CB Membranes

**Figure 1:**
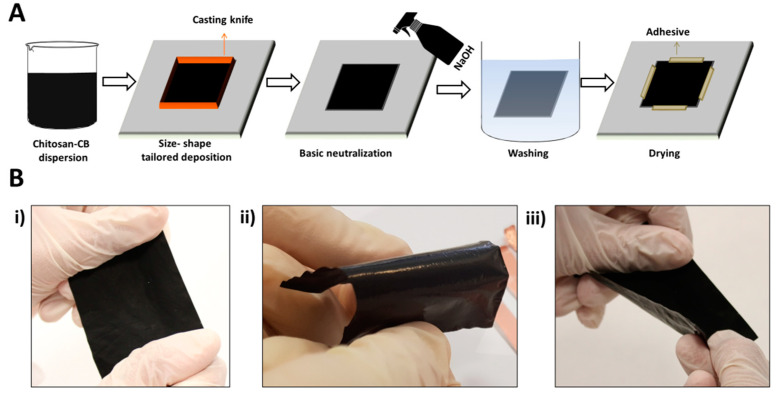
Flexible CH-CB membrane, 100% *w/w* of CB. (**A**) Fabrication strategy and (**B**) visual examination of the resilience to mechanical strain: (i) stretching, (ii) bending, and (iii) twisting.

The flexible CH-CB membrane fabrication strategy is shown in Figure 1. The slurry composed of CH and CB in different percentages was cast onto a glass surface using a Doctor Blade strategy by way of a stainless steel casting knife with 4 path. Once the film was dry, a NaOH solution was applied, and the membrane was removed from the glass surface, cleaned with water, and again fixed onto a glass surface for drying.

Using this simple synthesis procedure, a shape- and size-tailored and biocompatible membrane is developed with flexible (Figure 1) and conductive (Table 1) properties thanks to the presence of CH and CB. In addition, the CB-CH ratio has been optimized to ensure the mechanical properties of the developed membranes. In order to study the effect of the addition of CB on the conductive capacity of the membrane for the further development of bioelectrodes, different weight percentages of CB were used in the preparation of the CH-CB slurry. In addition, a membrane composed only of CH was also prepared for comparison. Thus, 25%, 75%, 100% and 200% CB with respect to CH were developed using the previously described methodology in order for them to be tested in terms of conductivity, charge transfer resistance, and mechanical stability for their further use in bioelectrode development for wearable applications. Five different membranes of each composition were developed, each one showing good reproducibility of the synthesis procedure and electrical properties.

### 3.2. Characterization of CH-CB Membranes

The electrochemical, surface, and mechanical properties of the developed membranes have been evaluated using scanning electron microscopy (SEM), 4-point resistivity measurements, micromechanical assays, and electrochemical impedance spectroscopy.

**Figure 2:**
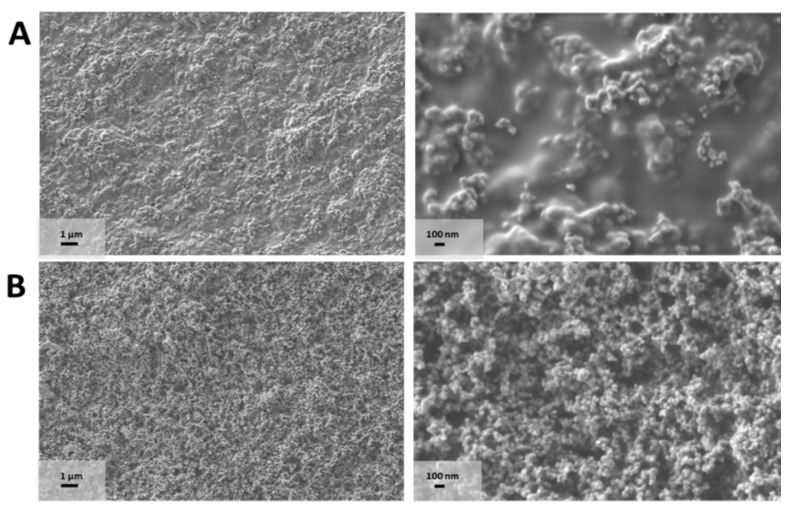
CH-CB membrane FESEM images of (**A**) 25 and (**B**) 100 w/w% CB recorded at 5 K × (**left**) and 30 K × (**right**).

The various CH-CB membranes were morphologically characterized using field emission scanning electron microscopy (FESEM). Figure 2 displays the FESEM images of the 25 and 100% *w/w* CB-CH membranes. In the FESEM images, the spherical morphology of CB particles and their nanometrical size with a diameter below 100 nm can be observed, which indicate the nanomaterial-based composition of the membrane. The membranes present a much higher developed surface area as opposed to the structures observed when the membranes were prepared without CB using graphite (Figure S1). The increase in the homogeneous distribution of the CB particles in the membranes is also notable as long as the CB percentage is higher, leading to a homogeneous carbon-containing fractal-looking matrix. The effect of the increase in CB content was also evaluated by FESEM for additional membranes containing 50%, 75%, 150% and 200% *w/w* of CH-CB (Supplementary Information, Figure S2), confirming that, below 50% weight of CB, the CB particles are less cohesive, leaving the CH polymer matrix visible. Therefore, CH presence is more notable in the membranes prepared with 25% *w/w* of CB due to the lower content of CB. As a result, it is possible to distinguish the polymeric matrix in these membranes. In contrast, when the CB percentage is above 75%, only a CB particle “porous” network can be observed.

This observation is interesting for the subsequent immobilization of enzymes on the surface of the membranes in order to develop bioelectrodes for BFCs and biosensors. The greater presence of activated carbon in the membrane will favor immobilization strategies that require previous functionalization of the carbonaceous support [36,39]. On the other hand, membranes with a lower proportion of CB will favor immobilization strategies governed by interactions from functionalities of the polymeric matrix. In this case, the amino functionalities present in the CH structure could be used for enzyme immobilization purposes [38,40], enabling two strategies for enzyme immobilization in the same support.

In addition to FESEM analysis, the 4-point probe method was used to evaluate the sheet resistivity of the membrane. The four different membranes prepared by changing the weight percentage of CB showed a decrease in sheet resistance, as long as CB content increases (Table 2). Furthermore, the variability of the values obtained for the membranes of five different batches allows us to confirm reproducibility of the membrane preparation procedure.

As expected, the most conductive membranes are those that have been prepared with a 200% CH-CB ratio. However, these membranes are fragile and brittle, thus preventing their application in bioelectrode development.

**Figure 3:**
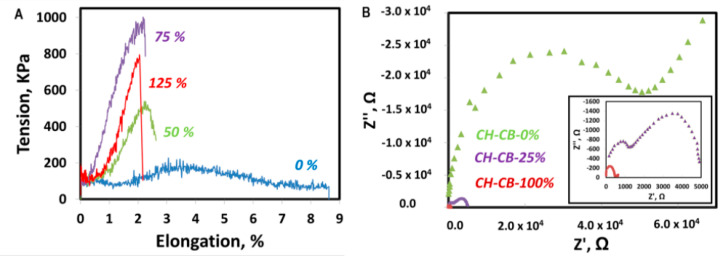
CH-CB membrane mechanical and charge resistance characterization. (**A**) Stress-strain curves of 0 (blue), 50% (green), 75% (purple), and 125% (red) w/w% CB CH-CB membranes. (**B**) Nyquist diagram of a CH membrane 0 (green), 25 (purple), and 100 w/w% CB-CH membrane (red). Inset: magnification of 25 and 100 w/w% CB-CH membranes.

The features relating to mechanical properties were evaluated under a force of 2000 N using a microassay module device. Before discussing the results obtained, it is worth mentioning tha, for CB weight percentages of 100% or lower, the membranes obtained showed suitable mechanical properties after drying for wearable sensing bioelectrode development. Only the membranes with two-fold CB with respect to CH were easily broken once the drying stage was finished. For this study, limit membranes were chosen as follows: 0% CB, representing the pure CH membrane; 50% CB, representing the higher limit for less cohesive CB particles in the composite; 75% CB, representing the limit where CB particles start to be cohesive; and 125% CB, representing the limit where CB content is higher than CH yet the material is no longer so brittle (Figure 3A).

After applying a 2000 N force, the elongation of the material was recorded until the membrane collapsed, leading to a drop in the applied force. In general, it can be observed that the increase in CB content is not beneficial for the mechanical properties of the membranes, and these properties deteriorate when CB content increases. The CH membrane without CB presents the behavior of a ductile plastic material, with an elastic zone with little slope and a more notable plastic zone that elongates until breakage with an elongation of around 9% [11]. However, once CB is incorporated into the membrane structure, it becomes more brittle with a higher slope in the elastic zone, while the plastic zone is barely present. For all the membranes with CB in their composition, breakage occurs at around 2% deformation. However, as the CB content increases, the membrane supports less maximum stress before breakage. Nevertheless, with a 125% weight of CB, the material breaks with less stress, which has already been observed during the manipulation of the membranes. Therefore, and as expected, it can be concluded that the incorporation of CB reduces, to some extent, the plasticity of the material because CB is intercalated between the polymeric chains, forming defects that prevent contact and interaction between the chains [34]. However, the mechanical properties of the selected membranes still fit with the envisioned application for wearable device development. Nevertheless, it is worth mentioning that it is not envisioned that the bioelectrodes developed in this work are to be implemented in devices such as elastic bands or gloves, which require high demanding flexibility properties, because they are not able to withstand high deformation forces. Despite this, the mechanical and electrical properties of the CH-CB membrane developed could be suitable for the development of other types of wearable devices, such as those for personal monitoring that are in direct contact with the skin (patches or tattoos), because they are not as highly exposed to heavy deformation forces. This has been evaluated by visual and hand examination, as in Figure 1, and with the results obtained in the evaluation of the mechanical and electrical properties (Figure 3 and Table 1).

Hence, the membrane of 100% weight of CB with respect to CH (CH-CB-100) will be used in this study due to its superior conductive capacity and its improved mechanical stability, taking into account the application envisioned for bioelectrode development. The conductivity values are similar to those provided by traditional graphite supports (Rs < 100 mΩ), and the CH-CB-100 membrane presents better conductive properties and mechanical stability than membranes where CB content is higher than CH content. Moreover, the membrane with a 25% w/w% CB (CH-CB-25) also appears to be suitable for enzyme immobilization and proves the suitability of different approaches based on COOH and NH_2_ groups. Thus, for Laccase biocathode development, CH-CB-25 has been selected because it presents sufficient conductivity and higher availability of amine groups, which allows the use of immobilization strategies for the oriented Laccase attachment, favoring direct electron transfer and taking advantage of the amino groups present in the CH matrix [38,39,40].

Electrochemical impedance spectroscopy (EIS) was used to test charge transfer resistance of the CH-CB-100 and CH-CB-25 membranes. This property is important in order to determine the efficiency of electron transfer on the electrode surface. In comparison with a pure CH membrane, the charge transfer resistance decreased more than 100-fold with respect to the membrane of CH without CB in its composition. Charge transfer resistance decreased from 40 kΩ for the pure CH membrane to 450 Ω for CH-CB-100. In the case of the CH-CB-25 membrane, charge transfer resistance decreased up to 10-fold when compared with the pure chitosan membrane with a measured value of 4500 Ω (Figure 3B).

### 3.3. Enzyme Attachment in CH-CB Membrane Characterization

The applicability of the CH-CB membrane for use as a bioelectrode in self-powered wearable devices has been tested by developing bioelectrodes for an enzymatic glucose BFC where covalent binding of the enzymes has been selected because it provides the best properties in terms of stability, something that is required for long term use of BFCs.

To this end, the enzymes GOx and Laccase have been used to develop an anode and a cathode of an enzymatic BFC using the developed membranes of 100 and 25 w/w% CB, respectively. In the case of the biocathode, covalent binding has been carried out using a strategy based on the formation of amide groups between the carboxylic groups of Laccase and the amine groups of CH, taking advantage of their higher availability in the membrane CH-CB-25 for enzyme attachment. By means of this strategy, Laccase is oriented in such a way that direct electron transfer is favored [39]. In the case of the bioanode, covalent binding has been carried out with an opposing strategy, using the carboxylic sites of the 100 w/w% CH-CB membrane after electrochemical oxidation and the amino groups of the enzyme to create amide covalent binding [36]. In this way, advantage is taken of the suitability of the membrane to address two different enzyme immobilization procedures, adapting it to different enzymes and thus making it possible to ensure a shorter distance from the active center to the conductive support, which will favor direct electron transfer between the enzyme and the electrode.

**Figure 4:**
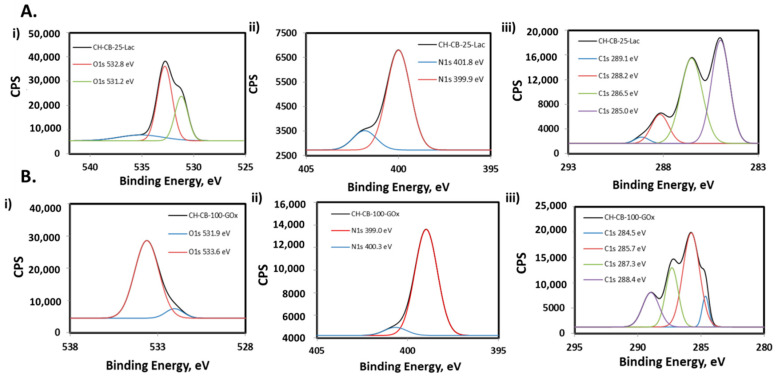
XPS spectra of (**A**) Laccasse CH-CB-25 membrane and (**B**) GOx CH-CB-100 membrane showing high resolution regions of (**i**) O1s, (**ii**) N1s, and (**iii**) C1s.

After enzyme attachment, the presence of the enzyme in the bioelectrode was tested using X-ray photoemission spectroscopy (XPS). Figure 4 shows the XPS high resolution spectra for the CH-CB-25 and CH-CB-100 membranes once Laccase and GOx have been covalently attached on their surface. Peak values and atomic percentages for C, N, and O, as well as the high resolution spectrum of the CH-CB membranes with and without enzymes, can be found in the Supplementary Information (Table S1 and S2 and Figures S3 and S4). In the case of the bioanode, electrochemical oxidation of the membrane as the first step of covalent binding of the enzyme has been confirmed by the increase of the atomic percentage of oxygen (from 18 to 21%) and analysis of the high-resolution regions of C1s and O1s where the presence of carboxylic acid groups is confirmed in the C1 region at 288 eV and the O1 region at 532 and 533 eV (Figure S3). Furthermore, the disappearance of these peaks as a consequence of the formation of new amide bonds proves the successful incorporation of GOx on the membrane. In addition, amide groups have also been detected in the analysis of the high resolution spectra of N1s (peak at 400 eV). Thus, results confirm that GOx has been efficiently immobilized on the surface of a CH-CB-100 membrane (Figure S3 and Table S1).

In the case of the biocathode, the enzyme Laccase has been attached to the bioelectrode based on a 25 w/w% CH-CB membrane. The efficient binding of Laccase has also been characterized by XPS. The experiments were performed for a membrane containing 25% *w/w* of CB before and after Laccase immobilization (Figure S4). The presence of Laccase in the membrane structure was first confirmed from the increase in N and O content from the atomic percentages obtained in the XPS measurements. Oxygen content increases from 15.8% to 25.1%, and the same occurs for nitrogen content, which increases from 2.0% to 5.9%. From the analysis of the high resolution regions, the presence of Laccase immobilized in the membrane can also be inferred. In the C1 high resolution spectrum for the membrane with Laccase, two new peaks are observed at 286.5 eV and 288.2 eV that were not present prior to Laccase incorporation (Table S2 and Figure S4). These peaks can be associated with carbon bound to N and or O in biomolecules in the form of amines or alcohols and to amide originating from a peptide bond. The same occurs with the O1 core level spectrum, where the peak at 531.2 eV, not present in the spectrum of the membrane without Laccase, could be associated with carbonyl groups from peptide bonds [40,51].

These results confirm efficient immobilization of both enzymes on the CH-CB membrane because the increase in the percentage of nitrogen and oxygen is observed as a consequence of the incorporation of the enzyme onto its surface and the changes observed in the high resolution regions of C1s, N1s, and O1s related to membrane surface functionalities.

### 3.4. Bioelectrodes: Cathode and Anode Enzyme Activity Evaluation

Once covalent binding had been carried out, catalytic activity of the enzymes was tested in response to glucose for energy harvesting and biosensing using CH-CB-100 for anode preparation and CH-CB-25 for the cathode development. For this purpose, Laccase and GOx-based CH-CB membrane bioelectrodes were first tested separately in a three-electrode cell configuration.

Biocathode enzymatic activity was tested by means of cyclic voltammetry and chronoamperometry. Cyclic voltammetry was recorded in the absence and the presence of oxygen to test enzymatic activity. Cyclic voltammetry measurements in oxygen and nitrogen confirm the presence of the immobilized Laccase on the surface of the bioelectrode membrane because the onset of oxygen reduction by Laccase is observed at potentials close to 700 mV. Chronoamperometry shows a decrease in current (increase in absolute value) when oxygen is introduced into the system, which proves the presence of immobilized Laccase on the surface of the electrode (Figure S5). However, the currents obtained are quite low, which could be due to the fact that the pH of the working electrolyte (pH = 7) is far from pH 4–5, the optimum pH range for Laccase activity [52].

On the other hand, the immobilization of GOx on the bioanode was evaluated by cyclic voltammetry in PBS in the absence of O_2_. In this case, the modified electrode presents a pair of oxidation-reduction peaks due to the redox process that occurs in the active center of the enzyme (Figure S5C). This test has been carried out at different scanning speeds in order to characterize the electrochemical behavior of the GOx-based bioelectrode. Thus, at a scanning speed of 4 mV s^−1^, the pair of redox peaks corresponding to the active center of the enzyme appear at potentials of −0.36 and −0.48 (vs. Ag/AgCl), and peak separation (E1/2) and formal potential (E0) were 122mV and −422 mV, respectively, which is in accordance with the values that can be found in the literature for the GOx enzyme [53]. This value is in accordance with values reported in the literature for GOx immobilization, which confirms the success of immobilization on this support [53]. Furthermore, the formal potential (E0) does not change as the scanning speed varies. Therefore, enzymatic activity is adequate and the process is highly reversible (Figure S5). Using the Laviron equation, the constant for the electron transfer (ks) has been calculated to provide a reference for the efficiency of electron transfer onto these surfaces. The ks value obtained for this system was 0.132 cm s^−1^, in the same range as other values reported, but lower due to the less conductive character of the electrode when compared to other previously reported values in the presence of CH. Thus, according to these calculations, 7 nmol cm^−2^ of GOx has been immobilized on the membranes [54,55].

**Figure 5:**
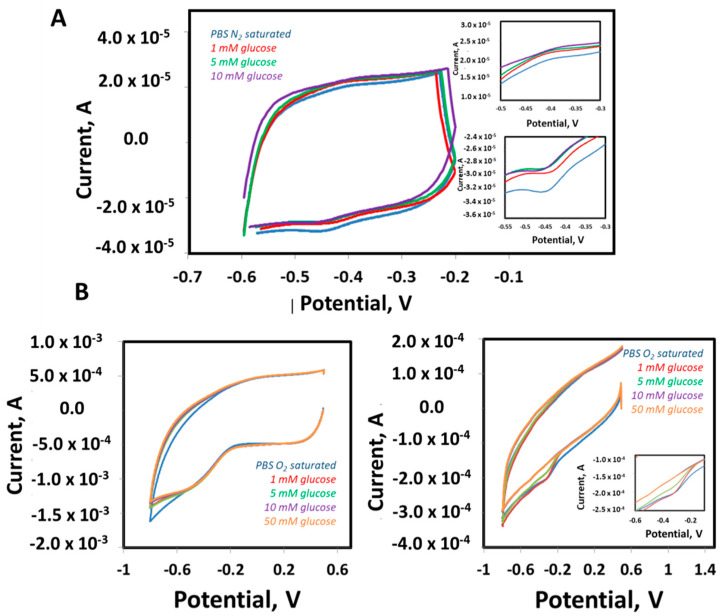
GOx-based bioanode enzyme activity evaluation. (**A**) Cyclic voltammperometry in PBS N2 saturated (blue) with different glucose concentrations: 1 mM (red), 5 mM (green), 10 mM (purple). Scan rate 4 mV s^−1^. (**B**) Cyclic voltammetry in PBS O_2_ saturated (blue) with different glucose concentrations: 1 mM (red), 5 mM (green), 10 mM (purple), 50 mM (orange). CH-CB electrode in the absence of GOx (left), CH-CB modified with GOx (right). Insets: magnification of reduction and oxidation peaks. Scan rate 75 mV s^−1^.

Furthermore, electrocatalytic activity was evaluated by the addition of glucose in the absence of O_2_ to allow active center visualization (Figure 5A) and in the presence of O_2_ (Figure 5B). In the case of the absence of O_2_, the peak corresponding to the oxidation of the active center increases or remains stable when glucose is added. However, the current corresponding to reduction of the active center decreases (becomes less negative) in a linear way as a function of the glucose concentration, progressively up to 5 mM and saturating at this concentration. The results obtained prove that the immobilized enzyme is participating in the catalysis of glucose and that the process is governed by direct electron transfer (in the absence of oxygen). These results also concur with those found in the bibliography for this kind of enzymatic modification [54,55].

In the case of O_2_ being present and in accordance with the behavior expected for the electrode modified with the GOx enzyme, the oxidation current of the active center remains stable, while the reduction current decreases (becomes less negative) along with the concentration of glucose, proving the perfect catalytic activity of the GOx (Figure 5B).

These results reinforce the biocompatibility properties of the CH-CB membrane as a suitable matrix for enzyme stabilization, because they keep their catalytic activity towards the target substrate once they have been immobilized in the self-standing membrane used as the bioelectrode support.

### 3.5. Applicability to Wearable Technologies: Glucose Biosensing and Energy Harvesting

**Figure 6:**
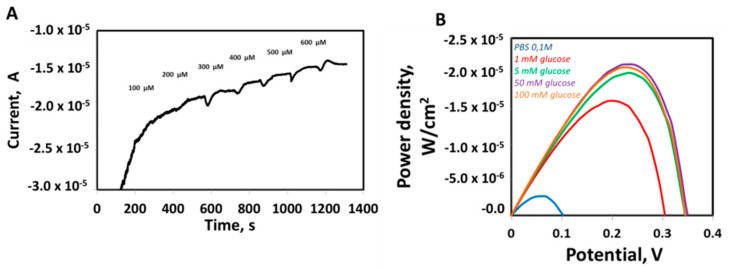
Enzyme-modified CH-CB membrane performance in sensing and energy harvesting applications. (**A**) Amperometry at −0.45 V for glucose detection in the presence of O_2_ using a CH-CB electrode modified with the GOx. Inset: calibration plot, linear range (100–600 µM). (**B**) Glucose BFC power density (GOx-based anode and Laccase-based cathode) in PBS 0.1M (blue) with different glucose concentrations: 1 mM (red), 5 mM (green), 50 mM (purple), 100 mM (orange).

Finally, sensing and energy harvesting capabilities were evaluated to test GOx activity and the suitability of CH-CB flexible and biocompatible membrane-based bioelectrodes for the development of wearable devices. Firstly, GOx-CH-CB membranes were evaluated as glucose sensors under the presence of O_2_. GOx catalyses the oxidation of glucose to H_2_O_2_ and D-gluconolactone under the presence of O_2_. In this context, O_2_ consumption in the system can be monitored for the indirect detection of glucose. Thus, increasing concentrations of glucose were evaluated in a range of 100 to 800 µM, with a linear concentration range of 100–600 µM (Figure 6A) and with a limit of detection (LOD) and quantification of 76 µM and 253 µM, respectively, taking into account the standard deviation of the intercept and the slope of the calibration curve as criteria for the calculations.

In addition, the electrochemical performance of the CH-CB membrane as a bioanode and biocathode of a glucose BFC was examined by assembling the GOx bioanode and Laccase biocathode, obtaining a suitable performance of the BFC under different glucose concentrations. Figure 6B shows the increase of the power density as a function of glucose concentration, with a characteristic bell-shaped curve where power density and open circuit voltage (OCV) are 21.3 µW·cm^−2^ and 0.35 V, respectively. Maximum power density is reached at 50 mM, and higher concentrations of biofuel do not yield an improvement in the power density obtained. It is also worth mentioning that the glucose BFC was able to deliver 15 µW cm^−2^, using just 1mM glucose as biofuel. These energy harvesting capabilities have been obtained by the use of a single pair anode-cathode with a geometric area of 0.6 cm^2^. This could be increased in further developments by the connection of various electrodes or modifications of the bioelectrodes in case maximum power inputs are required.

Thus, the suitability of CH-CB membrane-based working electrodes for glucose detection and energy harvesting is proof of the concept of the suitability of these membranes for wearable device development, confirming appropriate enzymatic activity after immobilization of the enzyme on CH-CB membranes.

In light of the results, as a final discussion, the performance of the developed membrane bioelectrodes is compared with the other reported systems in Table 2.

As can be seen in Table 2, the CH-CB membrane shows higher conductivity and similar properties in terms of sensing or energy harvesting purposes when compared with similar electrodes based on (bio)polymeric materials and carbon nanostructures. However, the CH-CB membrane is not superior compared with conductive polymers and doped membranes with extra nanomaterials or mediators, as was expected. Additionally, although it is suitable for sensing purposes, it does not improve the related works for this specific application. However, it is noteworthy in terms of the applicability of the system and the good response of the proposed CH-CB membrane to be used in energy harvesting applications. Specifically, the potential for energy harvesting is highlighted because a single pair anode-cathode with a geometric area of 0.6 cm^2^ has been used. Thus, the power input would be highly increased in further developments of this technology by the connection in series of more pairs of electrodes and/or the modifications of the bioelectrodes with biocompatible dopant components to maximize power inputs if required. Additionally, it is also worth mentioning the simplicity and sustainability of the fabrication process involving non-toxic reagents, which is essential for real applicability in the further development of wearable technologies.

## 4. Conclusions

In this study, a biocompatible membrane has been developed with suitable conductive and mechanical properties due to the combined presence of CH and CB. Various CH-CB amounts have been evaluated, with 25 and 100% *w/w* being selected as those most appropriate for bioelectrode development due to their mechanical and electrical properties, but also their features to allow two different strategies to maximize enzyme immobilization.

The CH-CB membranes synthesized in this study present conductivity values of 0.18 S cm^−1^ and 6.4 S cm^−1^ for membranes CH-CB25 and CH-CB100, respectively. In the case of the CH-CB25 membrane, its conductivity value is in the range of previously reported CH-carbon composite materials, as has been reviewed in the introduction part. However, the CH-CB100 membrane conductivity value is higher than those previously reported, which implies that the resistance barrier imposed by chitosan has been overcome thanks to the incorporation of a high weight percentage of CB. Furthermore, it involves several additional advantages when compared with previously reported membranes due to the simplicity and sustainability of the synthesis method that will not need any toxic reagents and solvents, extra energy input, or long reaction times. Additionally, it could be easily sized and shaped to suit any application, which makes it an extremely appropriate material for bioelectrode development, along with its ability to host specific enzymes due to its chemical and biocompatible properties. In addition, its suitability to be used in the development of bioelectrodes for glucose biofuel cells and biosensors has been proven thanks to the positive results obtained for glucose detection (LOD 76 µM in) and energy harvesting by means of a glucose biofuel cell (21.3 µW cm^−2^). These results confirmed the robustness and appropriateness of the membrane for this purpose. However, further development and research in nanoengineering and electronics should be carried out in order to miniaturize the membrane, enlarge the power density obtained, and improve the electronic interfacing for the efficient use of harvested energy to power small electronic devices.

## Figures and Tables

**Table 1:** CH-CB membrane electrical properties. Standard deviation is given for five different membranes. Electrical properties for amorphous carbon and graphite are also given [41].

CB w/w%	Thickness (µm)	ρ (Ω·sq−1)	Resistivity (Ω·cm)	Conductivity (S·cm−1)
25	26.3 ± 1.4	2100 ± 300	5.5 ± 0.7	0.18 ± 0.02
75	41 ± 2	51 ± 3	0.21 ± 0.01	4.7 ± 0.3
100	39.8 ± 1.0	37.4 ± 1.4	0.16 ± 0.01	6.4 ± 0.3
200	63.7 ± 1.3	19 ± 2	0.12 ± 0.02	8.5 ± 1.2
Amorphous carbon	-	-	0.05 − 0.08	20
Graphite	-	-	0.3	3.3

**Table 2:** Flexible and conductive bioelectrodes based on polymeric-carbon materials and glucose-related applications.

Bioelectrode Support Composition	Conductivity (S·cm^−1^)	Application		References
		Sensing (LOD, µM)	Energy Harvesting (Power Density, µW/cm^2^ and OCV, V)	
CH-CB-ITO	-	Tumor suppressor protein sensor (p53)	-	[15]
CH-RGO	0.01	-	-	[33]
CH-MWCNT	-	26	-	[37]
CH-CB-sponges	0.01	Strain sensor	-	[14]
PPY-CNT	-	-	1200/0.60	[42]
Au-GR-SWCNT	-	-	3.56/0.20	[43]
BP-MWCNT-CH-Nf	-	-	1600/0.60	[44]
Au-GRP-Co-CH	-	-	2200/0.60	[45]
CC-CH-TPP	-	-	1500/0.46	[46]
GCE-MWCNT-Mediator	-	0.30	32/0.35	[47]
GCH-NCNT	-	48	21/05	[48]
PANI-CNT-GCE	-	70	1120/0.78	[49]
GCE-CH	-	-	13/0.62	[50]
CH-CB-membrane	6.40	76	21.3/0.35	This work

CH: Chitosan; CB: Carbon Black; ITO: Indium Tin Oxide; RGO: Reduced Graphene Oxide; MWCNT: Multi-Wall Carbon Nanotubes; PPY: Polypyrrole; CNT: Carbon Nanotubes; GR: Graphene; SWCNT: Single-Wall Carbon Nanotubes; BP: Buckypaper; Nf: Nafion; GRP: Graphite; CC: Carbon Cloth; TPP: Tripolyphosphate; GCE: Glassy Carbon Electrode; GCH: Glutaraldehyde modified Chitosan; NCNT: Nitrogen-doped Carbon Nanotubes; PANI: polyaniline.

## Data Availability

Not applicable.

## Supplementary Materials

The following are available online at https://www.mdpi.com/article/10.3390/nano11082052/s1, Figure S1. SEM Images of a chitosan-graphite membrane. Figure S2. FESEM images of chitosan-CB membranes, Figure S3. Anode XPS spectra, Figure S4. Cathode XPS spectra, Table S1. Anode high resolution data from the XPS spectra. Table S2. Cathode high resolution data from the XPS spectra, Figure S5. Bioelectrode enzymatic activity.
