# Supplementary material for: Flexible and Conductive Bioelectrodes Based on Chitosan-Carbon Black Membranes: Towards the Development of Wearable Bioelectrodes
Source: Nanomaterials (Basel). 2021 Aug 12;11(8):2052. doi: 10.3390/nano11082052 (PMC8398670; doi:10.3390/nano11082052)
Supplement: Supplementary file 1 [file nanomaterials-11-02052-s001.zip › nanomaterials-1301249-supplementary.pdf]

nanomaterials

Supplementary Information

Flexible and Conductive Bioelectrodes Based on Chitosan-Carbon Black Membranes: Towards the Development of Wearable Bioelectrodes

Mireia Buaki-Sogó [1,*], Laura García-Carmona [1,*], Mayte Gil-Agustí [1], Marta García-Pellicer [1] and Alfredo Quijano-López [1,2]

[1] Instituto Tecnológico de la Energía (ITE), Avenida Juan de la Cierva, 24, 46980 Paterna, Spain; mayte.gil@ite.es (M.G.-A.); marta.garcia@ite.es (M.G.-P.); alfredo.quijano@ite.es (A.Q.-L.)
[2] Instituto de Tecnología Eléctrica, Universitat Politècnica de València, Camino de Vera s/n edificio 6C, 46022 Valencia, Spain
* Correspondence: mireia.buaki@ite.es (M.B.-S.); laura.garcia@ite.es (L.G.-C.); Tel.: +36-9-6136-6670 (M.B.-S.)

SEM of Chitosan-Graphite Membrane

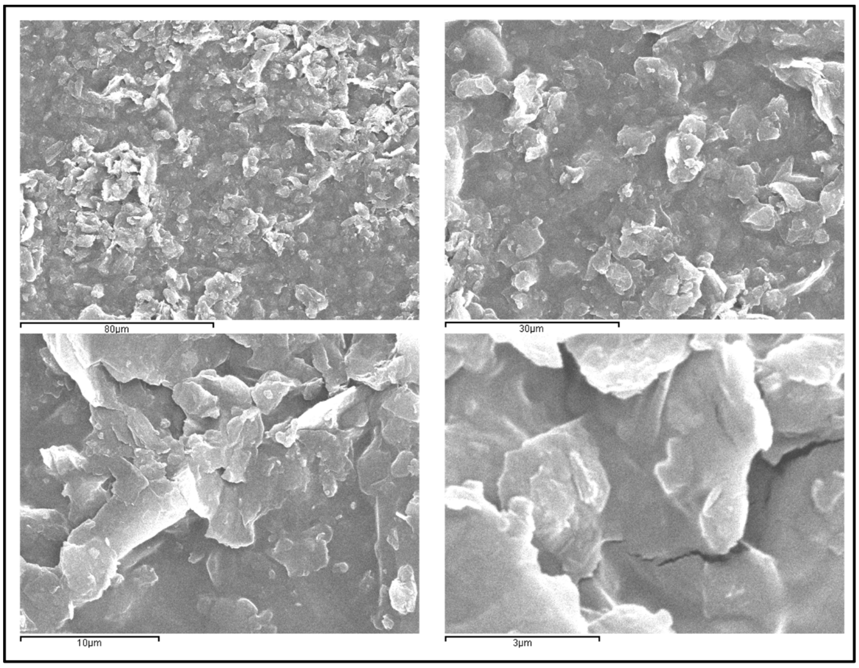

Figure S1. SEM Images of a chitosan-graphite membrane. Mass ratio of chitosan:graphite 1:3.

SEM of Chitosan-CB Membranes

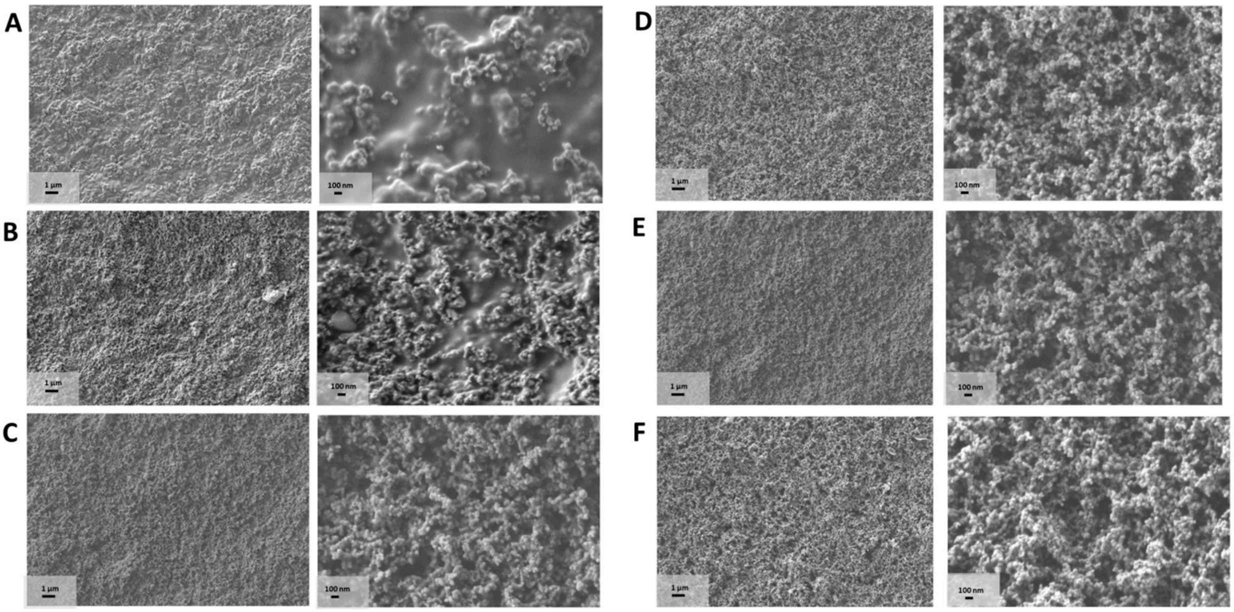

Figure S2. FESEM images of **(A)** 25, **(B)** 50, **(C)** 75, **(D)** 100, **(E)** 150 **(F)** 200 w/w% CB at 5 K × (left) and 30 K × (right).

X-ray Photoemission Spectroscopy for Enzyme Binding Characterization

In the case of the anode, in order to carry out covalent binding of the enzyme, electrochemical oxidation of the bioelectrode is required as the first stage to increase carboxylic groups as a subsequent glucose oxidase (GOx) binding site. To evaluate the correct immobilization process XPS spectra of CH-CB membrane, the oxidized CH-CB membrane and the GOx-oxidized-CH-CB membrane were compared (Figure S3).

In the general spectra obtained for each of the samples, we observed peaks corresponding to the atoms present in the composition of the chitosan-carbon black membranes: C (from chitosan and CB), N (from chitosan and the GOx enzyme) and O (from chitosan, carbon black and GOx). At first inspection, in the general spectra we can observe the main presence of C (295 eV) in the membrane without oxidation and without enzyme (blue line) and a considerable increase in the presence of N (400 eV) for the membrane once the GOx has covalently anchored(Figure S3). This proves the presence of amino acids and therefore the binding of the enzyme. From the general spectra, a relative quantification of the elements on oxidation states (functional groups) and the elemental quantification can be obtained in the high-resolution regions. The results obtained from the spectra in the high resolution regions for the three membranes indicated above are shown below (Table S1).

In the case of the cathode, a similar approach has been carried out to prove the presence of N groups after covalent binding of the enzyme without COOH formation since in this case it is not required for enzyme attachment (Figure S4 and Table S2**).**

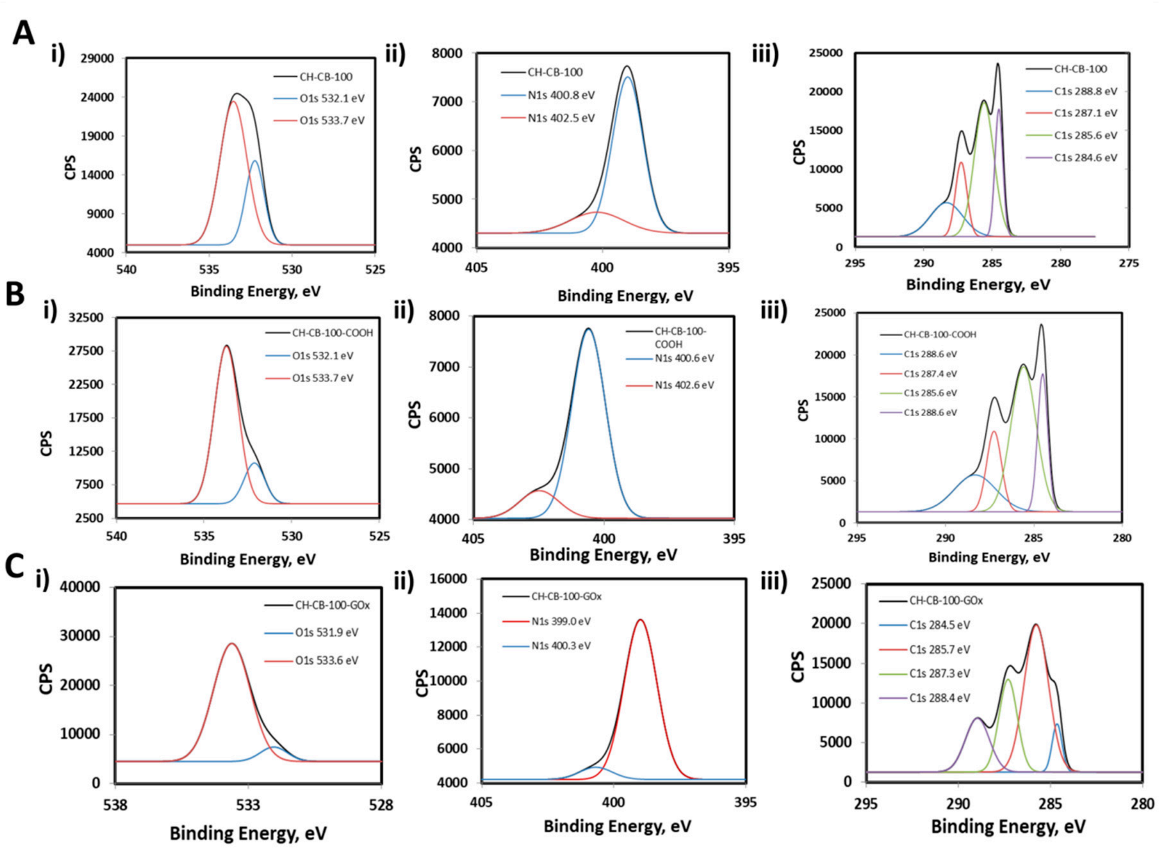

Figure S3. Anode XPS spectra. **(A)** CH-CB membrane (**B**) oxidized-CH-CB membrane and (**C**) GOx-oxidized-CH-CB membrane. i) O1s, ii) N1s and iii) C1s regions.

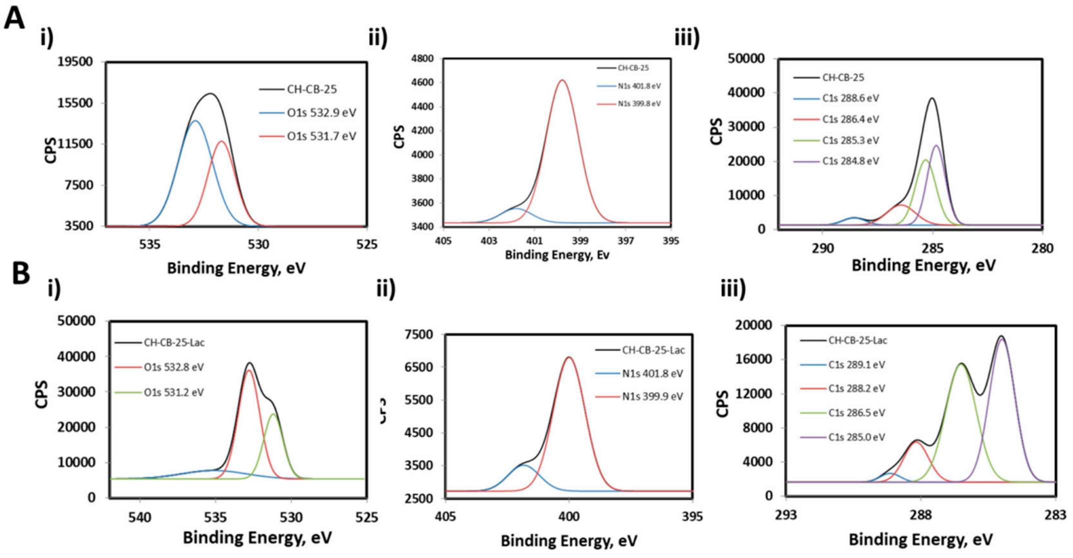

Figure S4. Cathode XPS spectra**. (A)** CH-CB membrane (**B**) Laccasse-CH-CB membrane. i) O1s, ii) N1s and iii) C1s regions.

Table S1 (anode) and Table S2 (cathode) show the binding energies for the different membranes obtained in the high resolution spectra and the functional groups to which they can be assigned together with the atomic percentages obtained. These percentages confirm effective oxidation of the membrane and the efficient immobilization within it of the enzyme glucose oxidase. This is observed in the increase of atomic percentages of oxygen, in the case of the

electrochemical oxidation treatment. In a similar manner, an increase in the percentage of nitrogen present in the membrane is observed as a consequence of the incorporation of the enzyme on its surface.

Table S1. Anode high resolution data obtained from the XPS spectra for CH-CB membrane GOx attachment.

Membrane	C1s	N1s	O1s	%C1s	%O1s	%N1s
CH-CB-100	284.6 eV (C-C/C=C/C-H)<br>285.6 eV (C-N / C-O)<br>287.1 eV (C-O-C / C-OH)<br>288.8 eV (C-N-C=O)	400.8 eV (NH-C=O)<br>402.5 eV (R-NH_3^+)	532.1 eV (C=O)<br>533.7 eV (C-OH/C-O-C)	78.3	17.8	3.8
CH-CB-100-COOH	284.6 eV (C-C/C=C/C-H)<br>285.6 eV (C-N / C-O)<br>287.4 eV (C-O-C/C-OH)<br>288.6 eV (C-N-C=O/ COOH)	400.6 eV (NH-C=O)<br>402.6 eV (R-NH_3^+)	532.1 eV (C=O)<br>533.7 eV (C-OH/C-O-C)	74.6	20.6	4.8
CH-CB-100-GOx	284.5 eV (C-C/C=C/C-H)<br>285.7 eV (C-N / C-O)<br>287.3 eV (C-O-C / C-OH)<br>288.4 eV (C-N-C=O/ COOH)	399.0 eV (R-NH_2)<br>400.3 eV (NH-C=O)	531.9 eV (C=O)<br>533.6 eV (C-OH/C-O-C)	70.3	19.8	9.9

Table S2. Cathode high resolution data obtained from the XPS spectra for CH-CB membrane and laccasse attachment.

Membrane	C1s	N1s	O1s	%C1s	%O1s	%N1s
CH-CB-25	285.0 eV (C-C/C=C/C-H)<br>285.6 eV (C=O/C-N)<br>286.7 eV (C-O-C / C-OH)<br>288.5 eV (C-N-C=O)<br>289.4 eV (O-C=O)	399.9 eV (NH-C=O)<br>401.8 eV (R-NH_3^+)	531.9 eV (C-OH)<br>533.1 eV (C-OR)	82.2	15.8	2.0
CH- CB-25-Lac	285.0 eV (C-C/C=C/C-H)<br>286.5 eV (C=O/C-N)<br>288.2 eV (C-O-C / C-OH)<br>289.0 eV (C-N-C=O/ O=C-O)	400.0 eV (NH-C=O)<br>401.8 eV (R-NH_3^+)	531.2 eV (C=O)<br>532.8 eV (C-OR/O=C-O)<br>535.8 eV ($H_2O_{(g)}$)	60.1	25.1	5.9

Bioelectrode Enzymatic Activity

For this purpose, the electrolyte solution was first purged in N_2 during 30 min and then the cyclic voltammogram was recorded. Subsequently, oxygen was introduced in the system during a further 30 min and the cyclic voltammetry was registered again. The chronoamperometry measurements were performed in the same electrolyte (PBS 0.1M), at a potential of 0 V and a scan rate of 1 mV/s. The system was purged with N_2 for 30 min while recording chronoamperomatry until the current was stabilized. Once a stable current in nitrogen was reached, pure O_2 was introduced into the system while recording the chronoamperometry F (Figure S5).

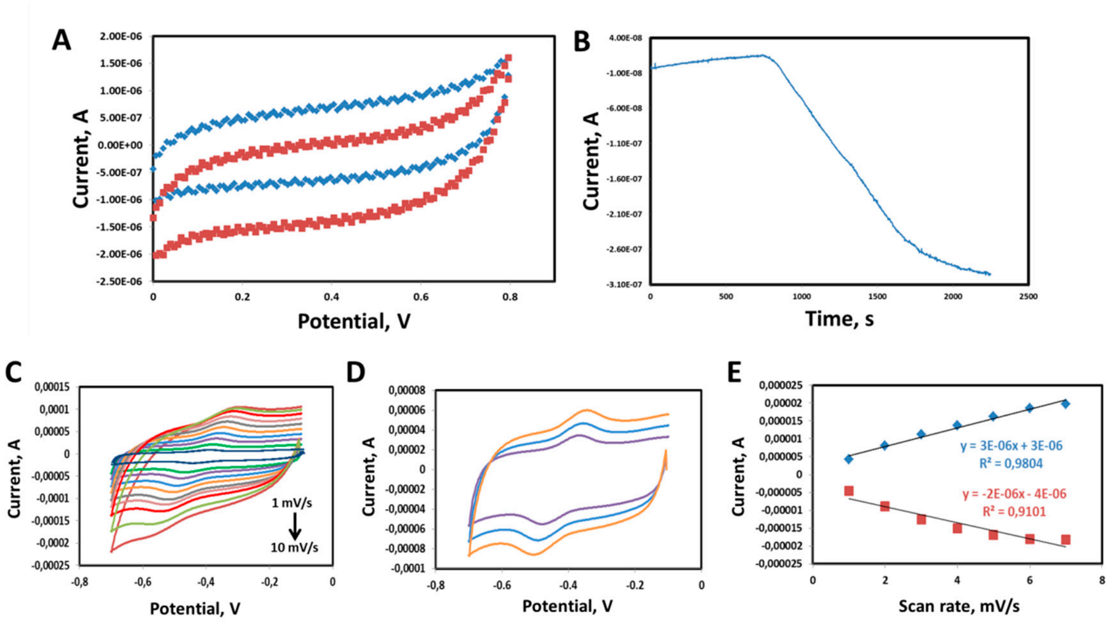

Figure S5. Bioelectrode enzymatic activity (A) Lacase-based biocathode cyclic voltammetry at 0V in PBS saturated with N_2 (diamond, blue) and O_2 (square, red) presence. **(B)** Lacase-based biocathode chronoamperometry. **(C)** GOx-based bioanode cyclic voltammetry in PBS saturated with N_2 speed from 1 to 10 mV s^{-1}. **(D)** Magnification of voltammets of 3, 4, and 5 mV/s. **(E)** GOx-based bioanode cyclic voltammetry. Relationship between peak current and sweep speed.
